# Ecosystem responses of shallow thermokarst lakes to climate-driven hydrological change: Insights from long-term monitoring of periphytic diatom community composition at Old Crow Flats (Yukon, Canada)

**DOI:** 10.1177/00368504231181452

**Published:** 2023-06-15

**Authors:** Wathiq J Mohammed, Lauren A MacDonald, Kathryn E Thomas, Ian McDonald, Kevin W Turner, Brent B Wolfe, Roland I Hall

**Affiliations:** 1 Department of Biology, 8430 University of Waterloo, Waterloo, Ontario, Canada; 2 Parks Canada Agency, Whitehorse, Yukon, Canada; 3 Department of Geography and Tourism Studies, 7497 Brock University, St. Catharines, Ontario, Canada; 4 Department of Geography and Environmental Studies, 8431 Wilfrid Laurier University, Waterloo, Ontario, Canada

**Keywords:** Vuntut National Park, Old Crow Flats Special Management Area, aquatic ecosystem monitoring, northern shallow lakes, hydroecology

## Abstract

Shallow waterbodies are abundant in Arctic and subarctic landscapes where they provide productive wildlife habitat and hold cultural and socioeconomic importance for Indigenous communities. Their vulnerability to climate-driven hydrological and limnological changes enhances a need for long-term monitoring data capable of tracking aquatic ecosystem responses. Here, we evaluate biological and inferred physicochemical responses associated with a rise in rainfall-generated runoff and increasingly positive lake water balances in Old Crow Flats (OCF), a 5600 km^2^ thermokarst landscape in northern Yukon. This is achieved by analyzing periphytic diatom community composition in biofilms accrued on artificial-substrate samplers at 14 lakes collected mostly annually during 2008–2019 CE. Results reveal that diatom communities at 10 of the 14 lakes converged toward a composition typical of lakes with rainfall-dominated input waters. These include six of nine lakes that were not initially dominated by rainfall input. The shifts in diatom community composition infer rise of lake-water pH and ionic content, and they reveal that northern shallow lake ecosystems are responsive to climate-driven increases in rainfall. Based on data generated during the 12 -year-long monitoring period, we conclude that lakes located centrally within OCF are most vulnerable to rapid climate-driven hydroecological change due to flat terrain, larger lake surface area, and sparse terrestrial vegetation, which provide less resistance to lake expansion, shoreline erosion, and sudden drainage. This information assists the local Indigenous community and natural resource stewardship agencies to anticipate changes to traditional food sources and inform adaptation options.

## Introduction

Arctic and subarctic permafrost landscapes support an abundance of shallow lakes and ponds that provide important wildlife habitat and sustain the cultural heritage and traditional land use of Indigenous communities.^1–4^ Due to their small volume relative to the surface area, northern shallow waterbodies are vulnerable to effects of climate warming, including rising air temperature and longer thaw season duration that increase evaporative water losses, and shifts in the amount and form of input from precipitation (snow and rain) and permafrost thaw.^5–13^ Such hydrological changes can alter biogeochemical cycling, water chemistry, habitat availability, and the structure and function of biological communities.^14–21^

Benthic algae play important roles in nutrient cycles, energy flow, and food web structure of shallow lakes.^22^ Diatoms, a group of eukaryotic algae that possess a siliceous frustule (outer casing), often dominate the phytobenthos and play key roles in the function of shallow freshwater ecosystems.^23^ Diatom communities are typically diverse, and individual taxa possess narrow ecological optima, which enables diatoms to respond to a wide range of environmental changes.^24,25^ This includes water chemistry and habitat availability caused by alterations in basin hydrology.^24–27^ These attributes have fostered the broad use of diatoms in biomonitoring programs to track environmental changes in aquatic ecosystems.^28–30^ Moreover, the composition of diatom communities in periphytic habitat and surface sediment captures an integrated signal of environmental changes over time and space within a lake, which serves to assess the vulnerability of shallow northern lakes to complex interactions among climate, hydrological processes, catchment features, and in-lake processes.^19,21,31^

**Figure 1.**
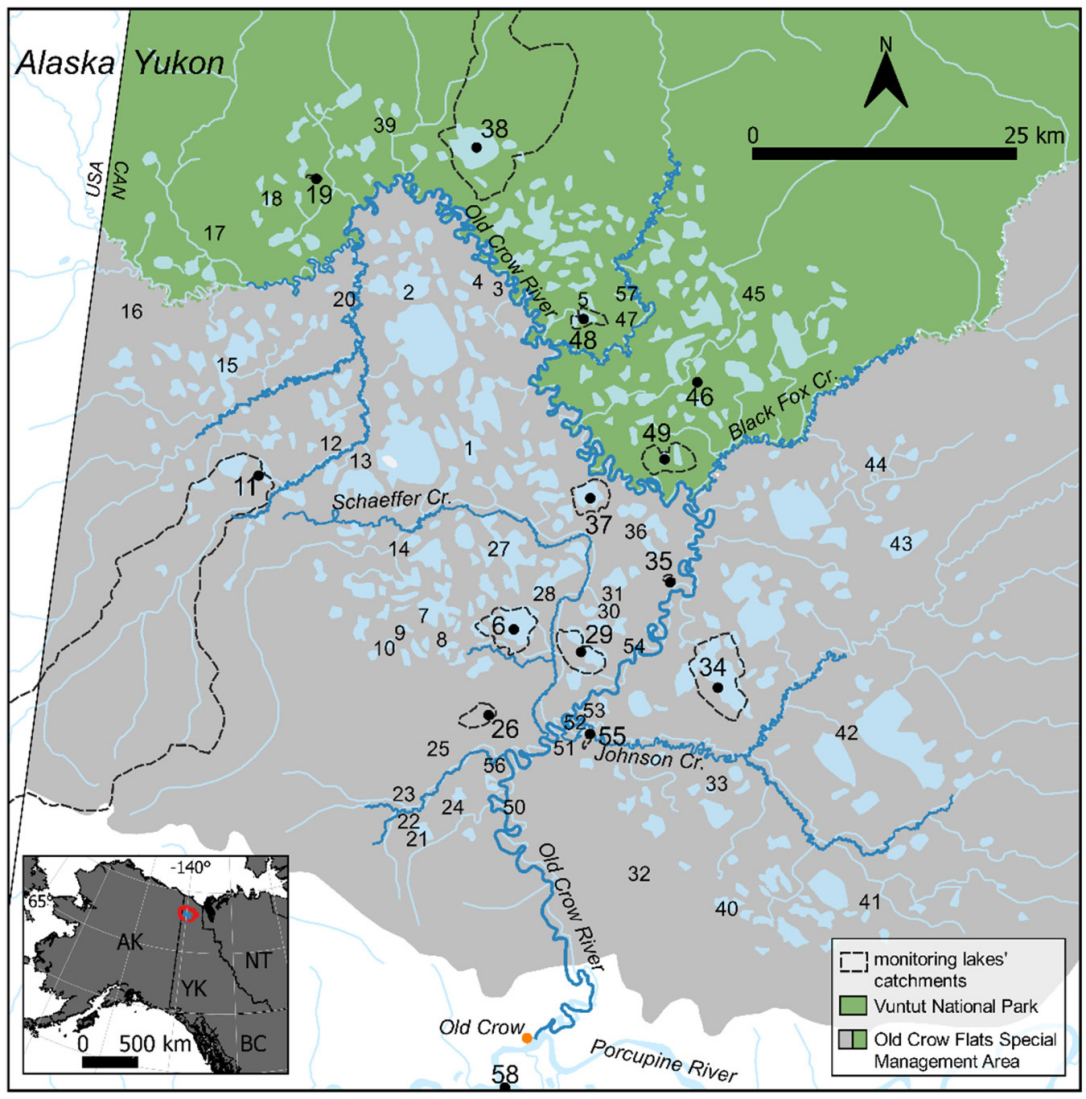
Map of the study area showing locations of the town of Old Crow (orange dot), Yukon, and the study lakes within OCF, protected within the OCF Special Management Area (grey shaded), including VNP (green shaded). The study lakes are numbered, and the 14 LTM lakes are distinguished by a black dot, larger font, and dashed line delineating their catchment area. A small inset map (lower left corner) identifies the location of OCF relative to territorial and federal boundaries.

LTM: long-term monitoring; OCF: Old Crow Flats; VNP: Vuntut National Park.

Old Crow Flats (OCF) is a 5600 km^2^ permafrost landscape in northern Yukon recognized as a Ramsar Wetland of International Importance for ecosystem services provided to wildlife and the Vuntut Gwitchin First Nation (VGFN) (Figure 1). Natural resources of OCF are protected within the OCF Special Management Area, including Vuntut National Park (VNP), and are cooperatively managed by the Vuntut Gwitchin Government (VGG), the Government of Yukon, Parks Canada Agency, and the North Yukon Renewable Resources Council. Concern has been expressed by the local community and natural resource stewards over climate-driven changes in the landscape, which include expansion, drainage and desiccation of lakes, proliferation of shrubs, and changes in river water levels.^11,32,33^ To address these concerns, a multidisciplinary International Polar Year (IPY) project was initiated in OCF in 2007 entitled “Yeendoo Nanh Nakhweenjit K’atr’ahanahtyaa (Environmental change and traditional use of Old Crow Flats in northern Canada: Looking after the land for the future).”^34^ One of the goals of this program was to develop and establish long-term monitoring (LTM) programs capable of tracking status, trends, and drivers of ecosystem change. Since 2007, partnerships among Parks Canada, VGG, and university researchers have advanced methods for LTM of the shallow, mainly thermokarst, lakes in OCF to track hydrological and limnological changes.^16,18,19,21,33,35–40^ Recently, increased input of rainfall runoff and, possibly, meltwater from degrading permafrost to lakes, have been identified based on water isotope monitoring of 14 lakes in OCF during 2007–2019,^39^ which increases the vulnerability of lakes to catastrophic drainage via outlet erosion.^16,36^

In this study, we use the analysis of periphytic diatom community composition in biofilms accrued on artificial-substrate samplers to track physicochemical and biological responses at the 14 LTM lakes in OCF during a 12-year period (2008–2019) associated with a rise in rainfall runoff and increasingly positive lake water balances.^39^ The findings improve knowledge of ecosystem responses of shallow thermokarst lakes to hydrolimnological changes and their vulnerability to climate change. This research contributes to an original goal of the IPY project, which was to “evaluate the impact of changes in the physical and biological environment on traditional food sources of the VGFN and community adaptation options.”^34^

## Study site

OCF, centered ∼55 km north of the village of Old Crow, is a dynamic wetland landscape that includes ∼8700 shallow (typically <3 m deep) and mainly thermokarst lakes amongst an assortment of terrestrial and riparian ecosystems (Figure 1).^41^ The lakes are perched above the Old Crow River, thus, the landscape exports waters from precipitation, runoff, and lake drainage. Fine-grained glaciolacustrine sediments and continuous permafrost underlie the low-relief landscape, which restricts the influence of sub-permafrost inflow and outflow on lake water balances.^42–45^

Based on a systematic sampling of a set of 57 lakes during 2007–2009, lakes across OCF have been categorized into those with snowmelt-dominated input and rainfall-dominated input from analysis and interpretation of water isotope composition.^18,19,21^ A third group of lakes was also distinguished, which receive either similar amounts of input from rainfall and snowmelt or substantial snowmelt runoff in spring and transition to rainfall-dominated during the thaw season.^18^ These “intermediate” lakes possess values of water chemistry variables, catchment features, and diatom community composition that overlap extensively with those of the rainfall- and snowmelt-dominated categories.^18,19,21^ Water chemistry analyses have revealed that lakes dominated by rainfall input possess higher lake-water pH, alkalinity, conductivity, and concentrations of major ions and dissolved inorganic carbon (DIC), and lower concentrations of major nutrients and dissolved organic carbon (DOC) compared to lakes dominated by snowmelt input due to differences in catchment vegetation and shoreline processes.^19,21^ Catchments of snowmelt-dominated lakes are dominated by trees and tall shrubs, which entrap wind-distributed snow and provide considerable snowmelt runoff during the ice-free season that elevates concentrations of nutrients and DOC via interaction with organic-rich soil, vegetation, and detritus.^18^ Higher ionic content, conductivity, and pH in rainfall-dominated lakes are considered to result from greater evaporative water loss from larger lake surface areas and the release of ions from erosion of sparsely vegetated shorelines.^16,18^ In parallel with the water chemistry study, periphytic diatom communities accrued on artificial-substrate samplers and in lake surface sediments were found to have higher relative abundance of *Achnanthidium minutissimum* and *Gomphonema angustum* in rainfall-dominated lakes, whereas snowmelt-dominated lakes have higher relative abundance of *Rossithidium pusillum*, *Sellaphora pupula, Eunotia bilunaris,* and *Tabellaria flocculosa*.^19,21^ These differences demonstrate the potential for periphytic diatom communities to serve as a biomonitor of aquatic ecological responses to hydrolimnological change.

The climate at Old Crow is continental, with cold winters (mean January air temperature = −31°C) and cool summers (mean July air temperature = 15°C). Mean annual precipitation is 257 mm, and the majority (157 mm) falls as rain during the thaw season, as reported by the Environment and Climate Change Canada meteorological station at Old Crow airport during 1981–2006. The 2007–2019 period of long-term lake monitoring is characterized by longer thaw season duration than the 1981–2006 average (147 vs. 141 days) and winter air temperature has been rising since 2013.^39^ For years of available data, average total thaw season precipitation (rainfall) during 2007–2019 (187 mm) is greater than the 1981–2006 mean (146 mm) recorded at the Old Crow airport, and total thaw season precipitation recorded within OCF by VNP during 2007–2016 is one standard deviation above the 1981–2006 mean. The average winter snow water equivalent during 2007–2019 is close to the 1981–2006 mean (120.1 and 103.8 mm, respectively). The years 2009 and 2011 stand out as anomalies when winter precipitation is well above average (153 and 360 mm, respectively).

For this study, 13 of the 14 LTM lakes were selected from the set of 57 lakes across OCF that were sampled three times per thaw season (typically early June, late July, and September/October) for water isotope composition and water chemistry during 2007–2009, and for the composition of periphytic diatoms accrued on artificial-substrate samplers in 2008 and 2009 (Figure 1). OCF 58 (Mary Netro Lake) was added in 2010 as the 14th LTM lake because it holds cultural significance for Vuntut Gwitchin residents in Old Crow and is boat accessible. As described by Tondu et al.,^40^ the 14 LTM lakes are well distributed across OCF and, at the time of selection, captured a representative range of hydrological conditions and catchment characteristics based on observations and isotope data obtained during 2007–2009. Five of the LTM lakes were classified as rainfall-dominated (OCF 6, 29, 37, 38, and 49), three as snowmelt-dominated (OCF 11, 55, and 58), and six as intermediate (OCF 19, 26, 34, 35, 46, and 48), based on the estimated isotope composition of input waters (δ_I_)^16^ and following lake classification methods outlined in Mohammed et al.^21^ The LTM lakes are typically shallow, except for OCF 55 where the depth exceeds 5 m, and they span a range of surface area (0.02 to 12.67 km^2^) and catchment area (0.28 to 395.19 km^2^; Supplemental Table S1). The catchments of the LTM lakes often include small portions of barren ground (1% to 8% of the catchment area), consisting of exposed rock, sand, and fire scar. An exception is OCF 6 (Zelma Lake), where barren areas covered almost half (43%) of the catchment, following a sudden catastrophic lake drainage event that occurred in June 2007.^40^ Substantial terrestrial land cover change has subsequently occurred, and as of 2017, tall willow shrub vegetation covered ∼31% of the former lake area, which stabilized hydrological and limnological conditions as snowpack increased.^37^ Based on paleolimnological evidence and aerial images, OCF 48 experienced a drainage event in ∼1989 and has since been refilled.^46,47^ Paleolimnological evidence for OCF 46 suggests increased thermokarst activity began in the 1970s and may have been followed by a drainage event.^47^ OCF 11 also has notably unique catchment characteristics with dense willow shrub surrounding the lake that suggests previous higher water levels, but remote sensing images suggest surface water levels have remained relatively stable since 1951.^11^

## Methods

### Sample collection and laboratory analyses

During 2007–2009, the LTM lakes were visited three times per year (typically early June, late July, and September/October) as part of the 57-lake spatial dataset. Since 2010, they have been visited at least twice per year (early June and September/October) within the LTM program, with additional visits in July conducted in 2015, 2016, and 2019. We use water isotope data from late July 2008 and 2009 to classify lakes into hydrological categories following methods presented in Mohammed et al.,^21^ as well as water isotope data from consistent sample collections in early June and September/October during 2007–2019.^39^ Water chemistry data utilized in this study were collected during visits in late July 2008 and 2009.^18,19,21^ Measurements of water chemistry at the LTM lakes since 2009 have occurred sporadically.^36^

The use of artificial-substrate periphyton samplers began in 2008, which were deployed in early June shortly after ice-off and retrieved in September/October shortly before ice-on. In 2015, inclement weather prevented the retrieval of periphyton samplers; thus, diatom data are missing for that year. Occasionally, periphyton samplers were not retrieved from some lakes in some years due to logistical challenges (see Supplemental Table S1). Each artificial-substrate sampler consists of two groups of five polyethylene sheets suspended 25 cm below a wooden float at the lake's surface and anchored to the lake bottom.^31,37^ Upon retrieval from each lake, the individual polypropylene sheets were placed in individual sample containers and kept in the dark. The retrieved samples were preserved frozen until analysis, and one sheet was randomly chosen from each sampler and analyzed for diatom community composition (as taxon percent abundances), following methods for the preparation of microscope slides described in Mohammed et al.^21^ A Zeiss Axioskop II Plus compound light microscope and 1000× magnification (numerical aperture = 1.30) under oil immersion was used to enumerate at least 300 diatom valves per sample and identify them to the lowest attainable taxonomic level (typically species or lower) using references by Krammer and Lange-Bertalot^48^ and Lavoie et al.,^49^ and updated nomenclature available at the Diatoms of North America (diatoms.org) and Algaebase (algaebase.org) websites.

### Numerical analyses

Multivariate ordination of the spatial dataset of periphytic diatom community composition at 57 OCF lakes sampled during 2008 and 2009 was used to inform interpretations of temporal changes in diatom community composition and infer shifts in water chemistry at the 14 LTM lakes. Samplers were retrieved from 33 and 48 of the lakes in 2008 and 2009, respectively. These data were analyzed and reported for each year individually in a prior study by Mohammed et al.^21^ to explore interannual differences. Here, we combined all the data from 2008 and 2009 in a single ordination to inform the interpretations of limnological change associated with shifts in community composition at the 14 LTM lakes during 2008–2019. Numerical analyses included 38 diatom taxa that occurred in at least two lakes and attained a maximum relative abundance of ≥2% (Supplemental Table S2). Here, we used constrained ordination of the spatial dataset of periphytic diatom community composition and water chemistry variables obtained in 2008 and 2009 as a “template” for inferring temporal shifts in water chemistry from observed changes in diatom community composition at the 14 LTM lakes. Analysis of the combined 2008–2009 diatom dataset by (unconstrained) detrended correspondence analysis indicated the floristic gradient length is 2.9 standard deviation units, which suggests that either unimodal- or linear-based constrained ordination methods may be suitable.^50^ We used unimodal-based canonical correspondence analysis (CCA), in part because CCA produced a slightly higher species–environment correlation along the first axis than did linear-based redundancy analysis (RDA; 0.92 vs. 0.86, respectively). CCA was also used in the prior analyses of interannual variation of diatom communities in 2008 and 2009.^21^ Thus, the use of CCA increases comparability between the studies. RDA explained a slightly larger amount of variance in the diatom data than did CCA (29.8 vs. 22.7%), but both methods supported comparable interpretations of temporal change in diatom community composition and inferred limnological change at the 14 LTM lakes. The combined 2008–2009 spatial dataset of the sampled lakes was entered as active samples in the CCA, whereas diatom percent abundance data obtained from the 14 LTM lakes during 2008–2019 were entered passively (i.e. they did not influence the ordination axes or the sample and taxon scores of the spatial dataset). This allowed the interpretation of change over time in diatom community composition at the LTM lakes and inferred water chemistry in the context of the spatial variation of hydrolimnological conditions that existed across lakes of OCF in 2008 and 2009. Water chemistry variables that individually explain a significant amount of variation in diatom community composition among lakes (at alpha = 0.05, based on 499 random Monte Carlo permutations) were incorporated as explanatory variables in the CCA (Supplemental Table S3). Other variables, including lake surface area, oxygen isotope composition of input water (δ^18^O_I_),^16^ isotope-derived evaporation-to-inflow ratio,^16^ and concentrations of total nitrogen (TN), ammonia (NH_3_), potassium (K), and chlorophyll-*a*, were included passively to assess their associations with the active water chemistry variables. Ellipses were added to the CCA plots to identify portions of the ordination space occupied by sample scores of the rainfall-dominated, intermediate, and snowmelt-dominated lake categories and to identify temporal patterns of variation in diatom community composition at each of the 14 LTM lakes during the period of record (2008–2019). All ordinations were performed using the software CANOCO version 5, and the ellipses were generated using the two-dimensional-normal-based ellipses option to span 66% of the distribution of samples within each category.^50^

## Results

### Re-analysis of the 2008–2009 OCF lake dataset: a template for assessing temporal changes in the LTM lakes

**Figure 2.**
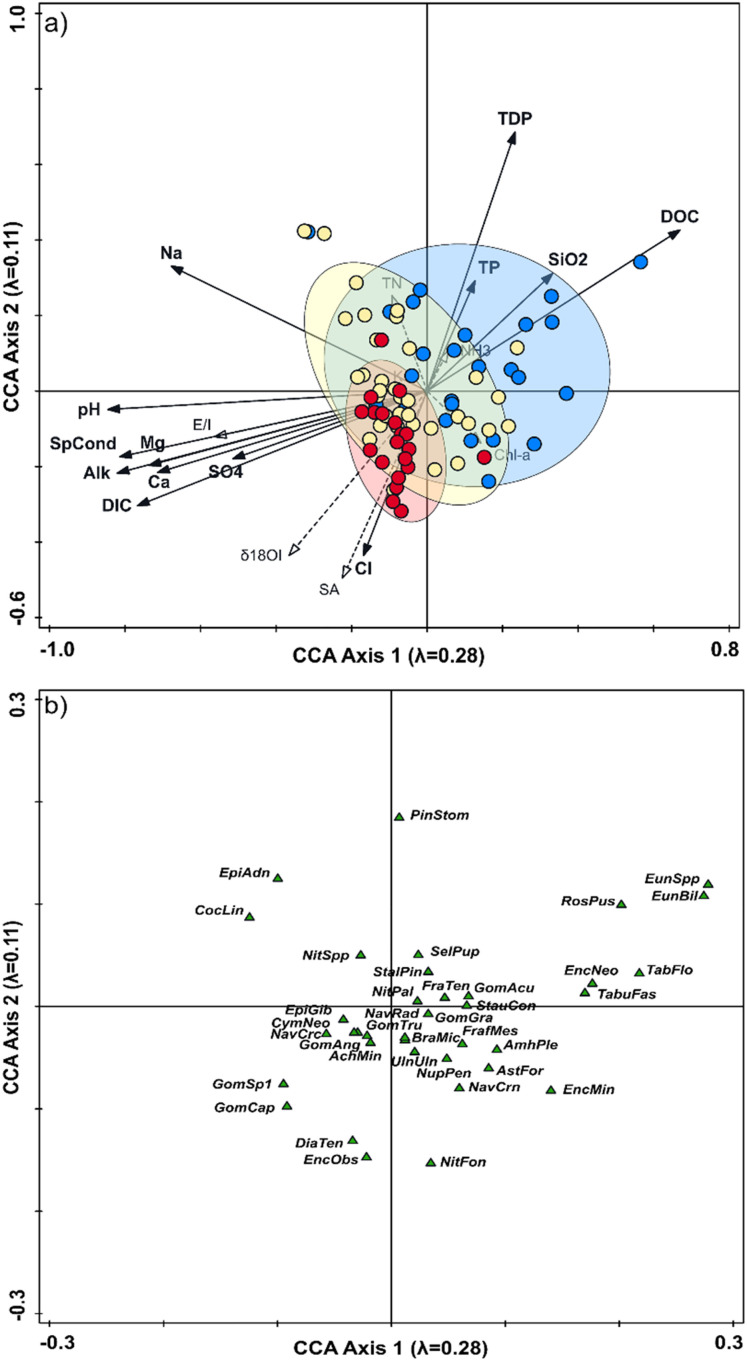
Graphs showing results of canonical correspondence analysis (CCA) of the water chemistry variables and diatom community composition (as taxon percent abundances) accrued on artificial-substrate periphyton samplers retrieved from 33 lakes in 2008 and 48 lakes in 2009 across Old Crow Flats. Water chemistry variables are shown as vectors and sample scores are presented as dots in panel (a), along with the 66% ellipses for the snowmelt-dominated lakes (blue), rainfall-dominated lakes (red), and intermediate lakes (yellow). Species scores are presented in panel (b), and full names of the abbreviated diatom taxon codes are reported in Supplemental Table S2. Codes used for the environmental variables in panel (a) are defined in Supplemental Table S3. The axes' lengths are longer in panel (a) than in panel (b) because the taxon scores are a weighted mean of the sample scores.

The first two CCA axes of the combined 2008–2009 spatial dataset explain 20.6% of the variance in periphytic diatom community composition and water chemistry variables (Axis 1 = 15.0%, Axis 2 = 5.6%; Figure 2). Sample scores of the snowmelt-dominated lakes are mostly situated to the right along CCA axis 1, whereas sample scores of the rainfall-dominated lakes are positioned to the left along axis 1 and mainly within the bottom left quadrant. These differences are illustrated by a small overlap of the sample scores from these lake categories. In contrast, sample scores of the intermediate lakes overlap extensively with those of the snowmelt- and rainfall-dominated lakes and are well scattered throughout the ordination space. Based on the environmental vectors, sample scores for the snowmelt-dominated lakes are associated with relatively high concentrations of total phosphorus (TP), total dissolved phosphorus (TDP), DOC, and silicon dioxide (SiO_2_), whereas rainfall-dominated lakes are associated with relatively high pH, alkalinity, and concentrations of major ions (Mg^+^, Na^+^, Cl^−^, and Ca^2+^) and DIC.

The composition of periphytic diatom communities varied systematically among the hydrological categories in the 2008–2009 spatial dataset (Figure 2). Taxa including *R. pusillum*, *S. pupula*, *E. bilunaris*, and *T. flocculosa* are relatively more abundant in several snowmelt-dominated lakes, as indicated by the position of their sample scores to the right along CCA axis 1, while they were mostly absent from the rainfall-dominated lakes (Supplemental Figure S1). *Diatoma tenuis* and *Encyonema obscurum* have higher relative abundance in the rainfall-dominated lakes than in the snowmelt-dominated lakes. The composition of periphytic diatom communities in the intermediate lakes overlapped with that of lakes in the other two categories, however, *Cocconeis lineata* was relatively more abundant in the intermediate category (Supplemental Figure S2).

### Temporal changes at the LTM lakes

**Figure 3.**
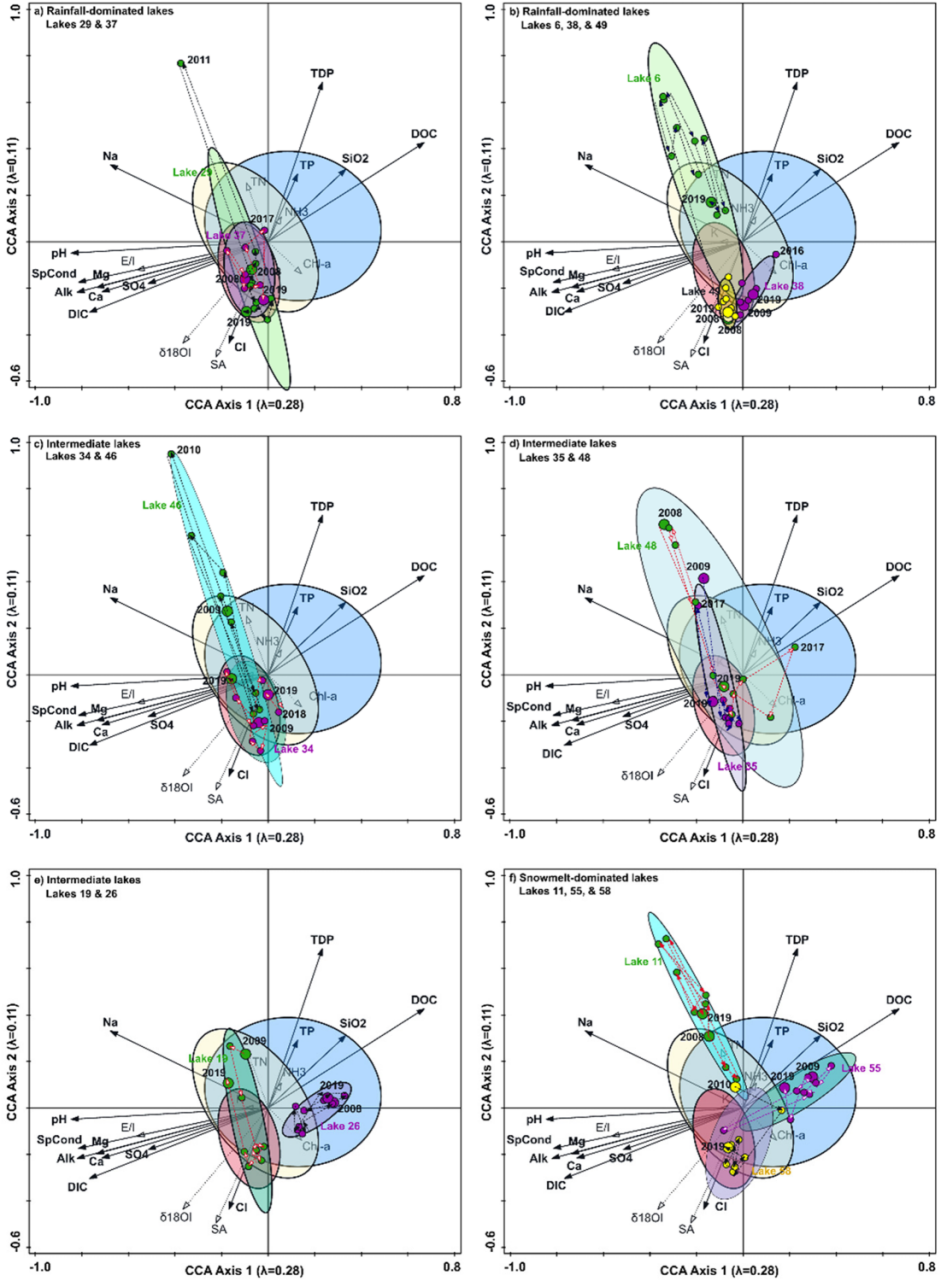
Graphs showing results of CCA based on the 2008–2009 spatial dataset of epiphytic diatom percent abundances and water chemistry variables across lakes of the OCF (from Figure 2), with the diatom percentage data from the 14 LTM lakes included passively. Red, yellow, and blue ellipses are the same as presented in Figure 2 (2D-normal-based 66% ellipses) and identify a representative range of samples scores from the rainfall-dominated, intermediate, and snowmelt-dominated lakes in the spatial dataset from 2008 and 2009, respectively. Also provided are the 66% ellipses about sample scores of each of the 14 LTM lakes to assess temporal patterns of change in sample scores (circles) and inferred water chemistry during the monitoring period (2008–2019). Panels (a) and (b) present the lakes that began the monitoring period in the rainfall-dominated category; panels (c), (d), and (e) present the lakes that began the monitoring period in the intermediate category; and panel (f) presents the lakes that began the monitoring period in the snowfall-dominated category. Codes used for the environmental variables are defined in Supplemental Table S3.

CCA: canonical correspondence analysis; 2D: two-dimensional; LTM: long-term monitoring; OCF: Old Crow Flats.

The CCA ordination of the 2008–2009 spatial dataset was used to track temporal changes in periphytic diatom communities and infer shifts in water chemistry at the 14 LTM lakes from 2008 to 2019 (Figure 3). If shifts in the composition of periphytic diatom communities track limnological changes caused by the documented increase in rainfall input to lake water balances,^39^ sample scores of lakes that were in the intermediate and snowmelt-dominated categories at the start of the monitoring period would be expected to shift leftward and downward to within the ellipse for the rainfall-dominated lakes in the CCA plots (Figures 2 and 3). This shift to rainfall-dominated lakes would be associated with declines in the relative abundance of *R. pusillum*, *S. pupula, E. bilunaris*, and *T. flocculosa* and a rise in the relative abundance of *A. minutissimum*, *G. angustum*, *D. tenuis, G. capitatum*, and *E. obscurum*. Such shifts also infer a decline in concentrations of TP, TDP, DOC, and SiO_2_ and an increase in pH, alkalinity, and concentrations of DIC and major ions.

Sample scores for four of the five LTM lakes that were classified as rainfall-dominated in 2008–2009 (OCF 29, 37, 38, and 49) spanned a narrow range located within or adjacent to the ellipse of the rainfall-dominated lakes in the CCA of the 2008–2009 spatial dataset, indicating that periphytic diatom community composition and inferred water chemistry remained relatively consistent over the course of the monitoring period (Figure 3(a) and (b)). *A. minutissimum* and *G. angustum* dominated the diatom communities in these four lakes for nearly the entire monitoring period, with their combined relative abundances occasionally exceeding 80% (Supplemental Figure S1). Diatom community composition in these lakes infers consistent and relatively high lake-water pH, alkalinity, and concentrations of DIC and major ions (Figure 3(a) and (b)). For these four lakes, the sample from 2011 at OCF 29 is the only exception to this pattern when the sample score shifted abruptly to the upper left quadrant of the CCA plot caused by an increase in the relative abundance of *C. lineata* from <5% in all other years to ∼95% in 2011. OCF 6, the fifth lake that began in the rainfall-dominated category, displayed a markedly different trajectory of change in diatom community composition during the monitoring period than the other four rainfall-dominated lakes (Figure 3(b)). The sample score for OCF 6 in 2008 was positioned in the lower left quadrant close to the other lakes in the rainfall-dominated category and associated with a high relative abundance of *A. minutissimum*. In 2009, however, assemblage composition became dominated by *C. lineata*, which resulted in a marked shift in sample scores to the upper left quadrant. After 2009, the relative abundance of *C. lineata* varied from 20% to 91% over the years, as illustrated by shifts in sample scores along axis 2. The declining relative abundance of *C. lineata* and increase of *A. minutissimum* and *G. angustum* resulted in a decline of sample scores along axis 2 to a location within overlapping ellipses of the snowmelt-dominated and intermediate lakes (Figure 3(b) and Supplemental Figure S1). The trajectory of sample scores for OCF 6 is largely orthogonal to the vectors for pH, alkalinity, and concentrations of major ions, TP, DIC, DOC, and SiO_2_, which suggests diatom communities responded to variation in other factors. OCF 6 experienced rapid, partial drainage in June 2007 caused by outlet erosion after a winter of above-average snowfall and intense rainfall in spring.^51,52^ More than 80% of the lake's volume was lost and surface area was reduced by nearly 43%.^32,35,50^

Sample scores for three of the six lakes that began in 2008 or 2009 in the intermediate category (OCF 35, 46, and 48) were positioned initially within the upper left quadrant and subsequently decreased along axis 2 to the lower left quadrant and fell within the ellipse for rainfall-dominated lakes by 2019 (Figure 3(c) and (d)). Diatom communities in these three lakes were dominated by *C. lineata* during the first two monitoring years (2008 and 2009), along with a substantial relative abundance of *A. minutissimum, G. angustum,Nitzschia palea, Epithemia adnata,* and *Fragilaria mesolepta*. The relative abundance of *C. lineata* varied markedly between the years 2008–2017 but declined noticeably in 2018–2019 when the relative abundance of *A. minutissimum* and/or *G. angustum* increased (Supplemental Figure S2). Sample scores for OCF 19 followed a similar temporal pattern as the above three lakes, but in 2019 they are positioned in the upper left quadrant of the CCA plots where ellipses of the intermediate and snowmelt-dominated lakes overlap due to increased relative abundance of *C. lineata* and *E. adnata*. Sample scores of all four of these lakes (OCF 19, 35, 46, and 48) displayed substantial interannual variation and oscillated between the ellipses of the intermediate and rainfall-dominated categories (Figure 3(c) to (e)). Overall, findings suggest diatom community composition at OCF 35, 46, and 48 converged toward that more typical of rainfall-dominated lakes by 2019, but the inferred change in water chemistry appears to have been more subtle based on the movement of sample scores orthogonal to most of the vectors of the water chemistry variables. Diatom community composition at lakes OCF 26 and OCF 34 showed a different temporal pattern than the other lakes that began in the intermediate category in 2008–2009 (Figure 3(c) and (e)). At OCF 34, diatom community composition was dominated by *A. minutissimum* and *G. angustum* in 2009 and sample scores fell within the ellipse of the rainfall-dominated lakes and subsequently remained relatively unchanged through the monitoring period (Figure 3(c)). In 2008 and 2009, OCF 26 was classified as an intermediate lake based on analysis of water isotope compositions, but the periphytic diatom communities were dominated by *T. flocculosa*, and sample scores fell within the ellipse of the snowmelt-dominated lakes (Figure 3(e)). During the monitoring period, diatom community composition remained relatively unchanged and indicative of relatively dilute ionic content and higher nutrient concentration typical of snowmelt-dominated lakes. Overall, during 2008–2019, diatom community composition shifted toward that typical of lakes in the rainfall-dominated category at five of the six lakes that began in the intermediate category. OCF 26 was an exception because it possessed a diatom community composition more reflective of snowmelt-dominated lakes in 2008 and remained relatively unchanged throughout the monitoring period.

Three of the LTM lakes began in the snowmelt-dominated category (OCF 11, 55, and 58) and they showed individual patterns of change in diatom community composition during the monitoring period (Figure 3(f)). Sample scores for OCF 58 shifted rapidly (2010–2012) to within the ellipse of the rainfall-dominated lakes. The floristic changes infer increases in lake-water pH, alkalinity, and concentrations of DIC and major ions, and decreases in concentrations of TP, TDP, DOC, and SiO_2_. At OCF 55, *R. pusillum* and *T. flocculosa* maintained consistent and moderate relative abundance, and the sample scores remained mainly within the ellipse of the snowmelt-dominated lakes during the monitoring period (Figure 3(f) and Supplemental Figure S3). One exception to this was in 2011 when an abrupt increase in the relative abundance of *A. minutissimum* shifted the sample score to within the ellipse of rainfall-dominated lakes. Sample scores for OCF 11 were consistently positioned within the upper left quadrant of the 2008–2009 “template” CCA plot, associated with the high relative abundance of *C. lineata* and *E. adnata*. During the monitoring period, sample scores for OCF 11 varied in a direction orthogonal to vectors for most water chemistry variables and displayed marked interannual variation and absence of gradual directional change toward the ellipse of rainfall-dominated lakes.

## Discussion

As aquatic biota are integrative of the complex interactions and multiple trajectories of ecosystem change,^53^ they are a useful monitoring tool for identifying ecosystem responses and vulnerability to the effects of climate warming.^4^ However, long-term biomonitoring records (i.e. ≥10 years) are rarely available from northern locations due to logistical challenges.^4,27,53–55^ To date, most biomonitoring programs in the North have been of short duration and have focused on rivers or on surveys of fish or abiotic variables in lakes rather than on long-term trends in the composition of algal communities in lakes.^54,56^ Long-term records of lake algal assemblages can provide important information and they have been shown to respond sensitively to climate-induced shifts in nutrients and hydrological connectivity.^56^ Studies have provided broad spatial analysis of diatom assemblage composition in northern lakes via a collection of surficial sediment samples, and some studies have assessed the composition of periphytic communities on naturally occurring substrates.^27,56–59^ Indeed, few studies have focused on long-term repeated biomonitoring of lakes in northern Canada. Exceptions include five consecutive years of benthic invertebrate sampling at lakes in the Mackenzie Delta, Canada, to assess relationships with hydrological connectivity,^60^ and monitoring of a suite of limnological and biological variables, including the composition of diatom communities in periphytic and planktonic habitats, every ∼3 years since 1983 at 45 lakes and ponds on Ellesmere Island.^14^ To our knowledge, our study is among these few long-term lake biomonitoring records in the Arctic and subarctic regions of Canada that exceed 10 years in duration.

**Figure 4.**
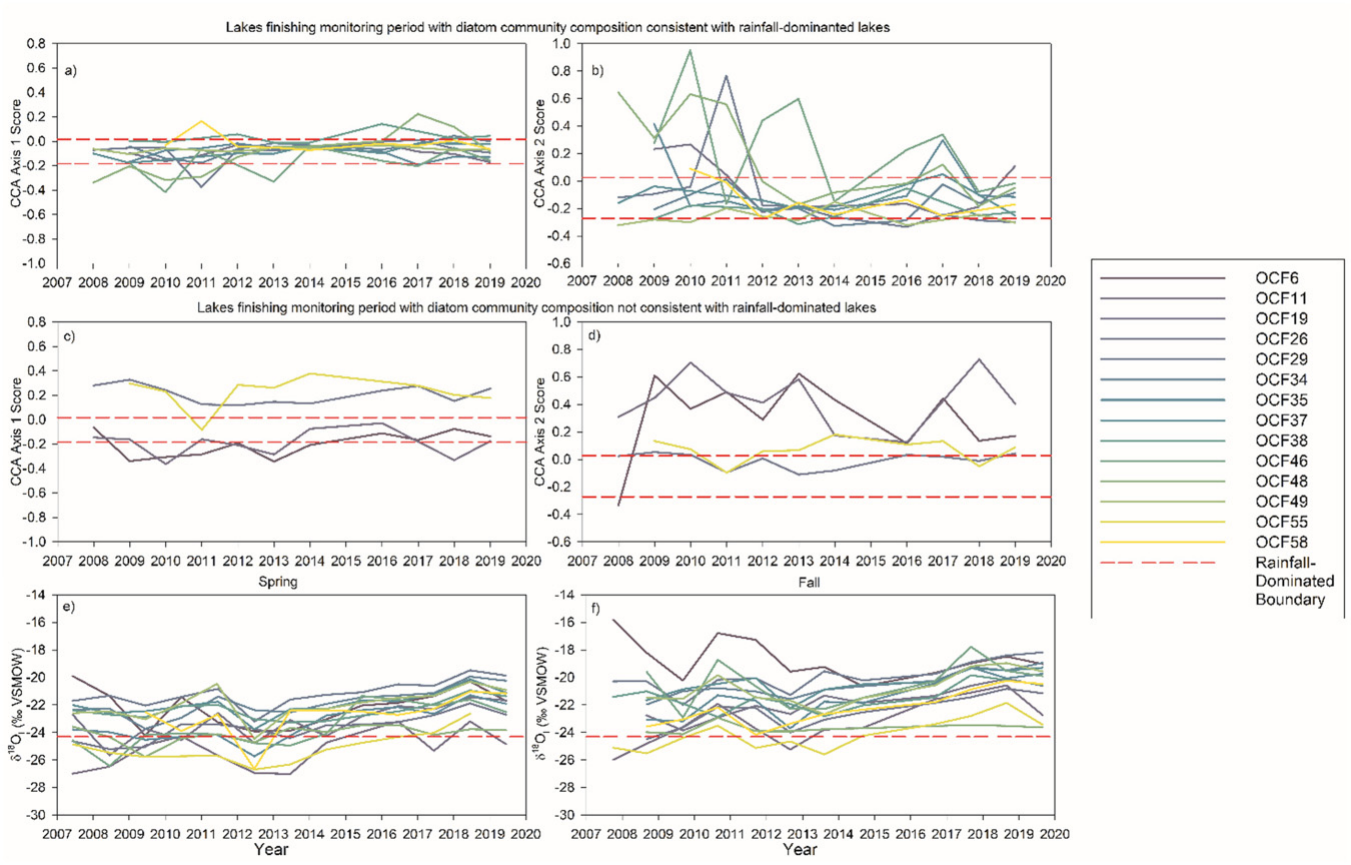
Graphs showing temporal patterns of variation in periphytic diatom community composition based on sample scores along axes 1 and 2 of the CCAs presented in Figure 3 and oxygen isotope composition of input waters (δ^18^O_I_) at the 14 LTM lakes in OCF during 2008–2019. Panels (a) and (b) show temporal patterns of variation in sample scores along CCA axis 1 and 2, respectively, for the monitoring lakes that possessed diatom community composition consistent with rainfall-dominated lakes in 2019. Panels (c) and (d) show temporal patterns of variation in sample scores along CCA axis 1 and 2, respectively, for the monitoring lakes that possessed diatom community composition not consistent with rainfall-dominated lakes in 2019. Panels (e) and (f) represent spring and fall input water isotope composition (δ^18^O_I_) of lakes during the monitoring period, and red dashed lines represent the threshold value for rainfall-dominated lakes as reported by MacDonald et al.^39^

CCA: canonical correspondence analysis; LTM: long-term monitoring.

Our analysis of periphytic diatoms accrued on artificial-substrate samplers reveals that community composition at 10 of the 14 LTM lakes (>70%) converged toward composition typical of lakes with rainfall-dominated input waters by 2019. This is based on sample scores for the LTM lakes in a CCA ordination which fell within or closely adjacent to the ellipse of the rainfall-dominated lakes in a spatial dataset from OCF lakes obtained in 2008–2009 (Figure 3). These include four of the five lakes that were dominated by rainfall input water at the beginning of the monitoring period (OCF 29, 37, 38, and 49), where diatom community composition remained relatively unchanged during the monitoring period. It also includes five of the six lakes that began in the intermediate category (OCF 19, 34, 35, 46, and 48) and one of the three lakes that began in the snowmelt-dominated category (OCF 58; Figure 3). Sample scores of these 10 lakes varied little along CCA axis 1 and mainly fell within the range of axis 1 values of rainfall-dominated lakes in the 2008–2009 spatial dataset (Figure 4(a)). Greater variation of sample scores for these 10 LTM lakes occurred along CCA axis 2, and they converged between 2014 and 2019 to values that fall within the ellipse for the rainfall-dominated lakes in the 2008–2009 spatial lake set (Figure 4(b)). Four of these 10 lakes (OCF 19, 34, 46, and 48) had the highest axis 2 scores from 2008 to 2013 and declined markedly thereafter along axis 2, due mainly to relatively high or rising percent abundance of the diatom taxa *A. minutissimum*, *G. angustum*, *G. capitatum*, *D. tenuis*, and/or *E. obscurum*. The patterns of change in diatom community composition captured along CCA axis 2 are consistent with rising δ^18^O_I_ values determined from measurements of lake water isotope composition, which demonstrate increasing input of rainfall and possibly permafrost thaw after 2013 (Figure 4(e) and (f)).^39^ The shifts in diatom community composition infer rising lake-water pH and alkalinity and rising concentrations of major ions (Mg^+^, Na^+^, Cl^−^, and Ca^−^) and DIC. Thus, the climate-driven increase in rainfall and associated runoff elicited ecological and water quality responses in a majority of the LTM lakes.

Four of the 14 LTM lakes (OCF 6, 11, 26, and 55) reveal patterns of change that are not consistent with a shift toward diatom communities typical of rainfall-dominated lakes (Figure 4(c) and (d)). OCF 6 drained suddenly and catastrophically in 2007, one year before diatom monitoring began, which resulted in a loss of over 80% of the lake volume and 40% of the surface area.^51^ Exposure of the former lake bottom promoted rapid encroachment of terrestrial shrubs, which increased the influence of snowmelt on the lake's water balance by entrapping wind-distributed snow.^36,52^ These post-drainage physical and ontogenetic changes at OCF 6 appear to have altered diatom community composition and inferred water chemistry in unique directions compared to other lakes that had not drained shortly before the onset of the monitoring program. Diatoms infer that TN and ammonia concentrations increased markedly during the early years of the monitoring program and remained elevated above values observed in 2008. This is consistent with sporadic mid-summer measurements of TN concentration reported for this lake by Turner et al.^36^ and may have been caused by higher rates of organic matter decomposition under conditions of shallower lake depth and pluvial runoff across exposed sediment after drainage.^61–63^ Hydrological conditions and water chemistry at OCF 6 became less variable seven years after the drainage event, once the shrub vegetation stabilized the former lakebed sediments.^36^ More consistent sample scores within the ellipse of the snowmelt-dominated lake category toward the end of the monitoring record suggest that the composition of the periphytic diatom communities has tracked these changes closely (Figure 3(a)). OCF 11 was identified as snowmelt-dominated in 2008 based on water isotope composition, but it displayed a similar trajectory of change in diatom community composition during the monitoring period as that observed at OCF 6, characterized by sample scores that were positioned within the upper left quadrant of the CCA and that shifted mainly along axis 2 due to relatively high and varying abundance of *C. lineata* and *E. adnata* (Figure 3(f)). Compared to the other lakes, OCF 11 has a particularly large catchment area (395.2 km^2^) and dense shrub vegetation adjacent to and within its basin (Figure 1 and Supplemental Table S1), which may have resulted in shifts in diatom community composition that reflect both an increase of rainfall runoff caused by a climatic trend^39^ and continued strong influence of snowmelt runoff from the dense shrubs in the catchment area. At the start of the monitoring record, OCF 26 was classified in the intermediate category and OCF 55 was in the snowmelt-dominated category, yet both lakes had diatom community compositions that consistently fell within the ellipse for snowmelt-dominated lakes during the monitoring period. The relative stability of diatom community composition and relatively high inferred concentrations of nutrients and DOC may be attributed to the small surface area of both lakes which normally reduces the influence of heating and evaporation by sunlight. Also, the catchment vegetation is dominated by tall shrubs at both lakes, which typically accumulates greater snowpack and reduces interannual variability of water balance and water chemistry.^16,33,40^ The greater depth of OCF 55 (>5 m) also may dampen the influence of hydrological processes on water chemistry and diatom community composition.

**Figure 5.**
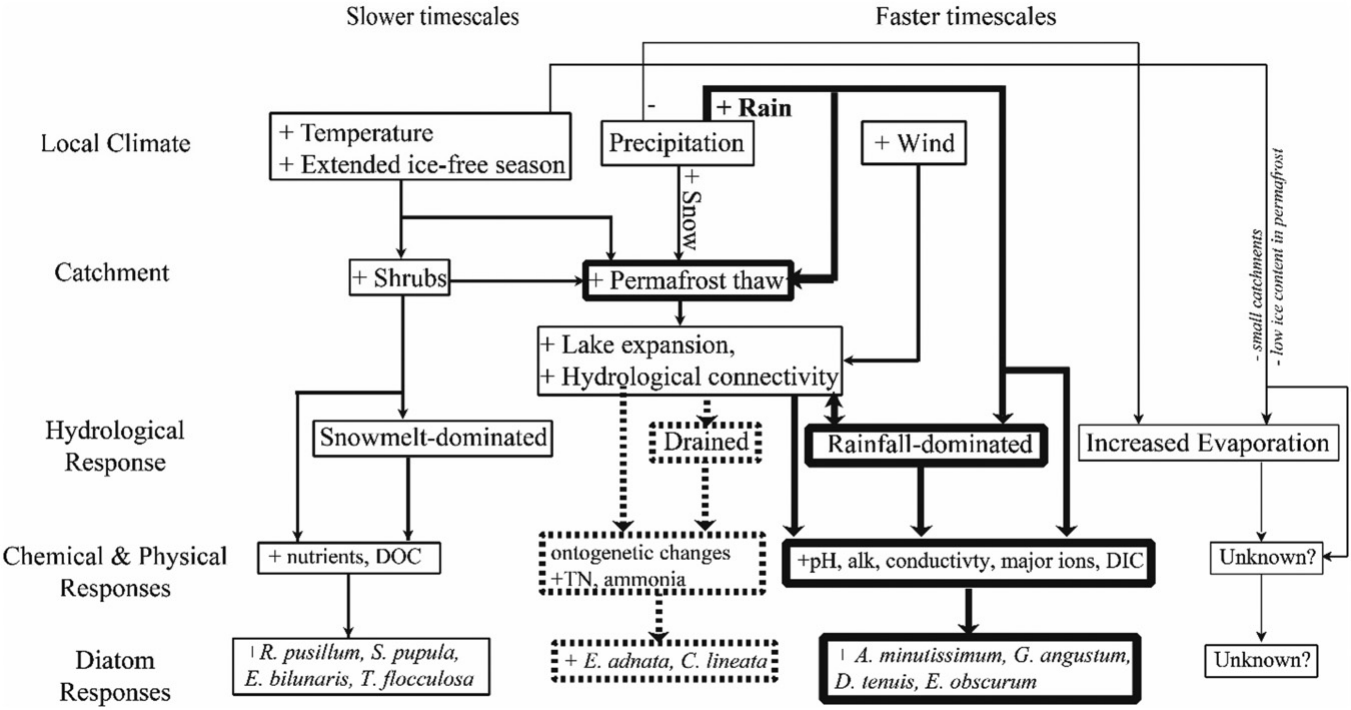
Schematic diagram representing the possible hydrological and limnological trajectories for lakes in Old Crow Flats (OCF) in response to climatic changes. Bold lines represent the pathways that are inferred to have become dominant in OCF during the past 13 years, and the dashed box around “Drained” represents a potential trajectory that may become more common. Limnological changes are separated into chemical and physical responses and responses of periphytic diatom community composition. Modified from MacDonald et al.^39^.

Water balance and ecological conditions of shallow lakes in Arctic and subarctic regions are influenced by interactions among several factors, which include air temperature, amount and form of precipitation, catchment vegetation, and permafrost dynamics.^11,64–67^ Consequently, complex and multiple hydroecological trajectories are possible under scenarios of future climate change, which challenge our ability to assess their vulnerability to greenhouse warming.^3,16,38,40^ At OCF, for example, Tondu et al.^40^ and Turner et al.^16^ postulated that climate warming will increase evaporative water losses and that greater shrub growth will increase the importance of snowmelt on lake water balance in some catchments. Analysis of 13-year-long hydrological monitoring records and meteorological data, however, has revealed a trend of increasingly positive lake water balance because of rising input from rainfall and potentially permafrost thaw which has outpaced losses by evaporation.^39^ Increasing summer rainfall has also been documented during the period spanning our study at the Cape Bounty Arctic Watershed Observatory in the Canadian High Arctic^54^ and across broad regions of the Arctic.^68^ Abrupt rise in lake water level caused by seasonal increases in input water increases the vulnerability of the lakes to sudden water loss by lateral lake drainage, as occurred in 2007 at OCF 6.^33,36,51^ Largely coincidental and corresponding shifts in diatom community composition demonstrate that northern shallow lake ecosystems are responsive to a climate-driven increase in rainfall and associated limnological changes. Moreover, long-term records of periphytic diatom community composition provide additional insight into trajectories of change in chemical and physical conditions and ecological responses, which improves our understanding of the underlying processes and the spatial and temporal scales at which they occur (Figure 5). For example, the rapid convergence of diatom community composition in 10 of the 14 lakes between 2010 and 2019 to that typical of lakes with rainfall-dominated input waters infers that pH, alkalinity, conductivity, and concentrations of major ions and DIC increased over a relatively short time period, likely in response to the erosion of shoreline substrates during lake expansion. Sudden lateral drainage also elicits a rapid response as substantial lake volume is lost and shrubs proliferate across the exposed lake bottom, but results in a marked rise in the concentration of total nitrogen and ammonia^36,52^ with an associated increase in the relative abundance of the diatoms *E. adnata* and *C. lineata*. These relatively fast responses likely occur at lakes located more centrally within OCF, where flat terrain, larger lake surface area, and sparse terrestrial vegetation provide less resistance to lake expansion, shoreline erosion, and sudden drainage. Ecosystem responses may occur at a slower pace at lakes located along the periphery of OCF, where hillslopes descend from the surrounding mountains, and catchment characteristics foster dominance of snowmelt input on their water balance and limnological conditions. Here, dense, tall vegetation and thick, organic-rich soils have developed over many decades to centuries, and long-lasting seasonal input of snowmelt runoff from the hillslopes permit input waters to accumulate substantial amounts of major nutrients and DOC on their route to the lakes. Denser shrub growth along the shorelines also reduces rates of shoreline erosion. These counterbalancing forces may overwhelm or, at least, slow the pace of influence from rising rainfall input. The knowledge gained from the LTM program has identified processes by which climatic shifts alter hydroecological conditions of lakes within OCF and some spatial features that may influence where change is likely to occur most rapidly, which can assist the local Indigenous community to anticipate changes to their traditional food sources and inform adaptation options.^34^ Within the relatively flat terrain of the OCF landscape, which supports an abundance of shallow thermokarst lakes, a climate-driven rise of rainfall runoff currently appears to be a main agent of change to lake water balance, water chemistry, and biota. In other Arctic and subarctic regions with more variable topography and deeper lakes, other expressions of climate change may alter biological communities and limnological conditions more strongly, including changes to ice regimes, thermal stratification, an inflow of sediment and chemical compounds (nutrients, carbon, and ions), and establishment of invasive species.^58,62,69^ Continued LTM of northern shallow lakes will undoubtedly further improve our understanding of key processes that influence their vulnerability to ongoing and future climatic shifts.

## Supplemental Material

sj-docx-1-sci-10.1177_00368504231181452 - Supplemental material for Ecosystem responses of shallow thermokarst lakes to climate-driven hydrological change: Insights from long-term monitoring of periphytic diatom community composition at Old Crow Flats (Yukon, Canada) Click here for additional data file. Supplemental material, sj-docx-1-sci-10.1177_00368504231181452 for Ecosystem responses of shallow thermokarst lakes to climate-driven hydrological change: Insights from long-term monitoring of periphytic diatom community composition at Old Crow Flats (Yukon, Canada) by Wathiq J Mohammed, Lauren A MacDonald, Kathryn E Thomas, Ian McDonald, Kevin W Turner, Brent B Wolfe and Roland I Hall in Science Progress

sj-xlsx-2-sci-10.1177_00368504231181452 - Supplemental material for Ecosystem responses of shallow thermokarst lakes to climate-driven hydrological change: Insights from long-term monitoring of periphytic diatom community composition at Old Crow Flats (Yukon, Canada) Click here for additional data file. Supplemental material, sj-xlsx-2-sci-10.1177_00368504231181452 for Ecosystem responses of shallow thermokarst lakes to climate-driven hydrological change: Insights from long-term monitoring of periphytic diatom community composition at Old Crow Flats (Yukon, Canada) by Wathiq J Mohammed, Lauren A MacDonald, Kathryn E Thomas, Ian McDonald, Kevin W Turner, Brent B Wolfe and Roland I Hall in Science Progress

## References

[1] Wrona FJ Prowse TD Reist JD , et al. Climate change effects on aquatic biota, ecosystem structure and function. Ambio 2006; 35: 359–369. 17256640 10.1579/0044-7447(2006)35[359:cceoab]2.0.co;2

[2] White D Hinzman L Alessa L , et al. The Arctic freshwater system: Changes and impacts. J Geophys Res 2007; 112: G04S–G054.

[3] Vincent WF Callaghan TV Dahl-Jensen D , et al. Ecological implications of changes in the Arctic cryosphere. Ambio 2011; 40: 87–99.

[4] Heino J Culp JM Erkinaro J , et al. Abruptly and irreversibly changing Arctic freshwaters urgently require standardized monitoring. J Appl Ecol 2020; 57: 1192–1198.

[5] Schindler DW Smol JP . Cumulative effects of climate warming and other human activities on freshwaters of Arctic and subarctic North America. Ambio 2006; 35: 160–168. 16944640 10.1579/0044-7447(2006)35[160:ceocwa]2.0.co;2

[6] Prowse TD Wrona FJ Reist JD , et al. Climate change effects of hydroecology of Arctic freshwater ecosystems. Ambio 2006; 35: 347–358. 17256639 10.1579/0044-7447(2006)35[347:cceoho]2.0.co;2

[7] Rowland JC Jones CE Altmann G , et al. Arctic landscapes in transition: responses to thawing permafrost. EOS Trans Am Geophys Union 2010; 91: 229–230.

[8] Bouchard F Turner KW MacDonald LA , et al. Vulnerability of shallow subarctic lakes to evaporate and desiccate when snowmelt runoff is low. Geophys Res Lett 2013; 40: 6112–6117.

[9] IPCC. Climate change 2014: The synthesis report (SYR) of the IPCC fifth assessment report (AR5). Cambridge: Cambridge University Press, 2014.

[10] Woo M Young KL . Disappearing semi-permanent snow in the high Arctic and its consequences. J Glaciol 2014; 60: 192–200.

[11] Lantz TC Turner KW . Changes in lake area in response to thermokarst processes and climate in Old Crow Flats, Yukon. J Geophys Res 2015; 120: 513–524.

[12] Roberts KE Lamoureux SF Kyser TK , et al. Climate and permafrost effects on the chemistry and ecosystems of high Arctic lakes. Sci Rep 2017; 7: 13292. 29038475 10.1038/s41598-017-13658-9 PMC5643399

[13] Jones BM Arp CD Grosse G , et al. Identifying historical and future potential lake drainage events on the western Arctic coastal plain of Alaska. Permafr Periglac Process 2020; 31: 110–127. 32194312 10.1002/ppp.2038 PMC7074070

[14] Smol JP Douglas MSV . Crossing the final ecological threshold in high Arctic ponds. Proc Natl Acad Sci USA 2007; 104: 12395–12397. 17606917 10.1073/pnas.0702777104 PMC1941480

[15] Medeiros AS Quinlan R . The distribution of the Chironomidae (Insecta: Diptera) along multiple environmental gradients in lakes and ponds of the eastern Canadian Arctic. Can J Fish Aquat Sci 2011; 68: 1511–1527.

[16] Turner KW Wolfe BB Edwards TWD , et al. Controls on water balance of shallow thermokarst lakes and their relations with catchment characteristics: A multi-year, landscape-scale assessment based on water isotope tracers and remote sensing in Old Crow Flats, Yukon (Canada). Glob Chang Biol 2014; 20: 1585–1603.

[17] Rühland KM Paterson AM Smol JP . Lake diatom responses to warming, reviewing the evidence. J Paleolimnol 2015; 54: 1–35.

[18] Balasubramaniam AM Hall RI Wolfe BB , et al. Source water inputs and catchment characteristics regulate limnological conditions of shallow subarctic lakes (Old Crow Flats, Yukon, Canada). Can J Fish Aquat Sci 2015; 72: 1058–1072.

[19] Balasubramaniam AM Medeiros AS Turner KW , et al. Biotic responses to multiple aquatic and terrestrial gradients in shallow subarctic lakes (Old Crow Flats, Yukon, Canada). Arct Sci 2017; 3: 277–300.

[20] Lamhonwah D Lafrenière MJ Lamoureux SF , et al. Evaluating the hydrological and hydrochemical responses of a high Arctic catchment during an exceptionally warm summer. Hydrol Process 2017; 31: 2296–2313.

[21] Mohammed WJ MacDonald LA Wolfe BB , et al. Use of artificial-substrate samplers to identify relations between periphytic diatom community composition and hydro-limnological conditions in shallow lakes of Old Crow Flats, Yukon Territory (Canada). Hydrobiologia 2021; 848: 4551–4567.

[22] Vadeboncoeur Y Steinman AD . Periphyton function in lake ecosystems. Sci World J 2002; 2: 1449–1468. 10.1100/tsw.2002.294 PMC6009390 12805932

[23] Smol JP . Pollution of lakes and rivers: A paleoenvironmental perspective. 2nd ed. Oxford: Blackwell Publishing, 2008.

[24] Julius ML Theriot EC . The diatoms: a primer. In: Smol JP Stoermer EF (eds) The diatoms: applications for the environmental and earth sciences. 2nd ed. Cambridge: Cambridge University Press, 2008, pp. 8–22.

[25] Smol JP Stoermer EF (eds) The diatoms: Applications for the environmental and earth sciences. Cambridge: Cambridge University Press, 2010.

[26] Cantonati M Lowe RL . Lake benthic algae: Toward an understanding of their ecology. Freshw Sci 2014; 33: 475–486.

[27] Kahlert M Rühland KM Lavoie I , et al. Biodiversity patterns of Arctic diatom assemblages in lakes and streams: Current reference conditions and historical context for biomonitoring. Freshw Biol 2020; 67: 116–140.

[28] Stevenson RJ Pan YD van Dam H . Assessing environmental conditions in rivers and streams with diatoms. In: Smol JP Stoermer EF (eds) The diatoms: Applications for the environmental and earth sciences. 2nd ed. Cambridge: Cambridge University Press, 2010, pp. 57–85.

[29] Kelly MG Chiriac G Soare-Minea A , et al. Use of phytobenthos to evaluate ecological status in lowland Romanian lakes. Limnologica 2019; 77: 125682.

[30] Kennedy B Buckley YM . Use of seasonal epilithic diatom assemblages to evaluate ecological status in Irish lakes. Ecol Indic 2021; 129: 107853.

[31] Wiklund JA Bozinovski N Hall RI , et al. Epiphytic diatoms as flood indicators. J Paleolimnol 2010; 44: 25–42.

[32] ABEK Co-op (Arctic Borderlands Ecological Knowledge Co-op). Community reports 2005–2006, https://nwtdiscoveryportal.enr.gov.nt.ca/geoportaldocuments/2005-06Community.pdf (2007, accessed 22 December 2022).

[33] Turner KW Wolfe BB Edwards TWD . Characterizing the role of hydrological processes on lake water balances in the Old Crow Flats, Yukon Territory, Canada, using water isotope tracers. J Hydrol 2010; 386: 103–117.

[34] Wolfe BB Humphries MM Pisaric MFJ , et al. Environmental change and traditional use of the Old Crow Flats in Northern Canada: An IPY opportunity to meet the challenges of the new northern research paradigm. Arctic 2011; 64: 127–135.

[35] Turner KW Edwards TWD Wolfe BB . Characterizing runoff generation processes in a lake-rich thermokarst landscape (Old Crow Flats, Yukon, Canada) using δ^18^O, δ^2^H and d-excess measurements. Permafr Periglac Process 2014; 25: 53–59.

[36] Turner KW Wolfe BB McDonald I . Monitoring 13 years of drastic catchment change and the hydroecological responses of a drained thermokarst lake. Arct Sci 2022; 8: 1094–1115.

[37] MacDonald LA Balasubramaniam AM Hall RI , et al. Developing biomonitoring protocols for shallow Arctic lakes using diatoms and artificial substrate samplers. Hydrobiologia 2012; 683: 231–248.

[38] MacDonald LA Wolfe BB Turner KW , et al. A synthesis of thermokarst lake water balance in high-latitude regions of North America from isotope tracers. Arct Sci 2017; 3: 118–149.

[39] MacDonald LA Turner KW McDonald IA , et al. Isotopic evidence of increasing water abundance and lake hydrological change in Old Crow Flats, Yukon, Canada. Environ Res Lett 2021; 16: 124024.

[40] Tondu JME Turner KW Wolfe BB , et al. Using water isotope tracers to develop the hydrological component of a long-term aquatic ecosystem monitoring program for a northern lake-rich landscape. Arct Antarct Alp Res 2013; 45: 594–614.

[41] Labrecque S Lacelle D Duguay CR , et al. Contemporary (1951–2001) evolution of lakes in the Old Crow Basin, northern Yukon, Canada: remote sensing, numerical modeling, and stable isotope analysis. Arctic 2009; 62: 225–238.

[42] Russell D Mossop D Goodfellow C . Remote sensing for waterfowl nesting and nesting habitat Old Crow Flats, Yukon Territory, Canada. In: PECORA IV conference, Sioux Falls, SD, October 10-12, 1978. Reston, VA: National Wildlife Federation.

[43] Smith SL Burgess M . Permafrost thickness database for northern Canada. Open File 4173, Geological Survey of Canada, 2002.

[44] Zazula GD Duk-Rodkin A Schweger CE , et al.Late Pleistocene chronology of glacial Lake Old Crow and the north-west margin of the Laurentide ice sheet. In: Ehlers J Gibbard PL (eds) Developments in quaternary sciences. Dordrecht, The Netherlands: Elsevier, 2004, vol. 2, Part B, pp.347–362.

[45] Roy-Léveillée P Burn CR . Permafrost conditions near shorelines of oriented lakes in Old Crow Flats, Yukon Territory. In: 63rd Canadian geotechnical conference and 6th Canadian permafrost conference, Canadian Geotechnical Society, Calgary, Alta, 2010, pp. 1509–1516. https://pubs.aina.ucalgary.ca/cpc/CPC6-1509.pdf

[46] MacDonald LA Turner KW Balasubramaniam AM , et al. Tracking hydrological responses of a thermokarst lake in the Old Crow Flats (Yukon Territory, Canada) to recent climate variability using aerial photographs and paleolimnological methods. Hydrol Process 2012; 26: 117–129.

[47] Bouchard F MacDonald LA Turner KW , et al. Paleolimnology of thermokarst lakes: a window into permafrost landscape evolution. Arct Sci 2017; 3: 91–117.

[48] Krammer K Lange-Bertalot H. Bacillariophyceae. In: Pascher A (ed.) Süsswasserflora von Mitteleuropa, Stuttgart, Germany: Gustav Fischer Verlag, 1986–1991, vol. 2, Bands 1-4 (pp. 876, 610, 596, 468).

[49] Lavoie I Hamilton PB Campeau S , et al. Guide d'identification des diatomées des rivières de l'Est du Canada. Quebec: Presses de L’Université du Quebec, 2008.

[50] ter Braak C Šmilauer P . *Canoco reference manual and user’s guide: Software for ordination (version 5)* . Ithaca, NY: Microcomputer Power, 2012.

[51] Wolfe BB Turner KW . Near-record precipitation causes rapid drainage of Zelma Lake, Old Crow Flats, Northern Yukon Territory. Meridian 2008; Spring: 7–12.

[52] Tondu JME Turner KW Wiklund JA , et al. Limnological evolution of Zelma Lake, a recently drained thermokarst lake in Old Crow Flats (Yukon, Canada). Arct Sci 2017; 3: 220–236.

[53] Mallory ML Gilchrist HG Janssen M , et al. Financial costs of conducting science in the Arctic: examples from seabird research. Arct Sci 2019; 4: 624–633.

[54] Beel CR Heslop JK Orwin JF , et al. Emerging dominance of summer rainfall driving high Arctic terrestrial-aquatic connectivity. Nat Commun 2021; 12: 1448. 33664252 10.1038/s41467-021-21759-3 PMC7933336

[55] Goedkoop W Culp JM Christensen T , et al. Improving the framework for assessment of ecological change in the Arctic: A circumpolar synthesis of freshwater biodiversity. Freshw Biol 2021; 67: 210–223.

[56] Lento J Goedkoop W Culp J , et al. State of the Arctic freshwater biodiversity. Akureyri, Iceland: Conservation of Arctic Flora and Fauna International Secretariat, 2019.

[57] Michelutti N Holtham AJ Douglas MSV , et al. Periphytic diatom assemblages from ultra-oligotrophic and UV transparent lakes and ponds on Victoria Island, and comparisons to other diatom surveys in the Canadian Arctic. J Phycol 2003; 39: 465–480.

[58] Smol JP Wolfe AP Birks HJB , et al. Climate-driven regime shifts in the biological communities of Arctic lakes. Proc Natl Acad Sci USA 2005; 102: 4397–4402. 15738395 10.1073/pnas.0500245102 PMC555516

[59] Rühland KM Hargan KE Jeziorski A , et al. A multi-trophic exploratory survey of recent environmental changes using lake sediments in the Hudson Bay lowlands, Ontario, Canada. Arct Antarct Alp Res 2014; 46: 139–158.

[60] Scott RW Tank SE Wang X , et al. Are different benthic communities in Arctic delta lakes distinguishable along a hydrological connectivity gradient using a rapid bioassessment approach? Arct Sci 2019; 6: 463–487.

[61] Watanabe S Laurion I Chokmani K , et al. Optical diversity of thaw ponds in discontinuous permafrost: A model system for watercolor analysis. J Geophys Res Biogeosci 2011; 116: G02003.

[62] Vonk JE Tank SE Bowden WB , et al. Reviews and syntheses: Effects of permafrost thaw on Arctic aquatic ecosystems. Biogeosciences 2015; 12: 7129–7167.

[63] Natali SM Watts JD Rogers BM , et al. Large loss of CO_2_ in winter observed across the northern permafrost region. Nat Clim Chang 2019; 9: 852–857. 35069807 10.1038/s41558-019-0592-8 PMC8781060

[64] Myers-Smith IH Forbes BC Wilmking M , et al. Shrub expansion in tundra ecosystems: dynamics, impacts and research priorities. Environ Res Lett 2011; 6: 045509.

[65] Derksen C Brown R . Spring snow cover extent reductions in the 2008–2012 period exceeding climate model projections. Geophys Res Lett 2012; 39: L19504.

[66] Lantz TC Marsh P Kokelj SV . Recent shrub proliferation in the Mackenzie Delta uplands and microclimatic implications. Ecosystems 2013; 16: 47–59.

[67] Arndt KA Santos MJ Ustin S , et al. Arctic greening associated with lengthening growing seasons in Northern Alaska. Environ Res Lett 2019; 14: 125018.

[68] Walsh JE Bigalke S McAfee SA , et al.Precipitation. In: Druckenmiller ML Thoman RL Moon TA (eds) Arctic report card. Washington, USA: National Oceanic and Atmospheric Administration, 2022. 10.25923/n07s-3s69.

[69] Saros JE Arp CD Bouchard F , et al. Sentinel responses of Arctic freshwater systems to climate: Linkages, evidence, and a roadmap for future research. Arct Sci 2023; 9: 356–392.

[70] Wager E Kleinert S . Responsible research publication: international standards for authors. A position statement developed at the 2nd world conference on research integrity, Singapore, July 22–24, 2010. In: Mayer T Steneck N (eds) Promoting research integrity in a global environment. Singapore: Imperial College Press/World Scientific Publishing, 2011, Chapter 50, pp. 309–316.

