# Supplementary material for: Ecosystem responses of shallow thermokarst lakes to climate-driven hydrological change: Insights from long-term monitoring of periphytic diatom community composition at Old Crow Flats (Yukon, Canada)
Source: Sci Prog. 2023 Jun 15;106(2):00368504231181452. doi: 10.1177/00368504231181452 (PMC10358572; doi:10.1177/00368504231181452)
Supplement: sj-docx-1-sci-10.1177_00368504231181452 - Supplemental material for Ecosystem responses of shallow thermokarst lakes to climate-driven hydrological change: Insights from long-term monitoring of periphytic diatom community composition at Old Crow Flats (Yukon, Canada) [file sj-docx-1-sci-10.1177_00368504231181452.docx]

**Supplemental Material 1 for “Ecosystem responses of shallow thermokarst lakes to climate-driven hydrological change: Insights from** **long-term monitoring of periphytic diatom community composition at Old Crow Flats (Yukon, Canada)”**

by Mohammed, Wathiq J, Lauren A. MacDonald, Kathryn E. Thomas, Ian McDonald, Kevin Turner, Brent B. Wolfe, Roland I. Hall.

**Temporal Patterns of Change in Composition of Diatom Communities Accrued on Artificial-Substrate Samplers in the 14 Long-term monitoring Lakes in Old Crow Flats (2008-2019)**

The following pages present a Table listing selected characteristics of the 14 long-term monitoring lakes and the years when artificial substrate samplers were successfully retrieved from them (Table S1), a Table with the diatom taxon names, authority names, and codes presented in the CCA ordination for the combined 2008-2009 data in Figure 2 and time-series graphs in Figure S1 (Table S2), and a Table presenting the summary statistics and P-values for each of the water chemistry variables presented in the CCA ordinations in Figures 2 and 3 (Table S3). Also included are a series of graphs showing temporal patterns of change in relative abundance of select diatom taxa accrued on artificial-substrate samplers during 2008-2019 in the long-term monitoring lakes (Figures S1-S3).

Table S1. Selected characteristics of the 14 long-term monitoring lakes in Old Crow Flats based on Tondu et al. (2013), and the list of years when artificial samplers were successfully retrieved from each lake during the monitoring period (2008-2019). The number of long-term monitoring lakes with samplers retrieved is also provided for each year of the study (bottom row).

| **Lake** | **Local Name** | **Original Category**  **in 2008-2009** | **Catchment Area (km^2^)** | **Lake Surface Area (km^2^)** | **Depth (m)** | **Periphyton Sample Retrieved? (Y/N)** | | | | | | | | | | | |
| --- | --- | --- | --- | --- | --- | --- | --- | --- | --- | --- | --- | --- | --- | --- | --- | --- | --- |
|  |  |  |  |  |  | **2008** | **2009** | **2010** | **2011** | **2012** | **2013** | **2014** | **2015** | **2016** | **2017** | **2018** | **2019** |
| **OCF 11** |  | Snow | 395.19 | 0.07 | 0.8 | Y | Y | Y | Y | Y | Y | Y | N | Y | N | Y | Y |
| **OCF 55** |  | Snow | 0.59 | 0.02 | >5.0 | N | Y | Y | Y | Y | Y | Y | N | Y | Y | Y | Y |
| **OCF 58** | Mary Netro Lake | Snow | 5.50 | 0.38 | 2.6 | N | N | Y | Y | Y | Y | Y | N | Y | Y | Y | Y |
| **OCF 19** |  | Intermediate | 0.58 | 0.11 | 0.9 | N | Y | Y | Y | Y | Y | Y | N | Y | Y | Y | Y |
| **OCF 26** |  | Intermediate | 5.21 | 0.42 | 1.7 | Y | Y | Y | Y | Y | Y | Y | N | Y | Y | Y | Y |
| **OCF 34** | Nèhtrùh Vavan | Intermediate | 29.16 | 6.11 | 1.5 | N | Y | Y | Y | Y | Y | Y | N | Y | Y | Y | Y |
| **OCF 35** |  | Intermediate | 0.80 | 0.14 | 1.1 | N | Y | Y | Y | Y | Y | Y | N | Y | Y | Y | Y |
| **OCF 46** |  | Intermediate | 0.28 | 0.12 | 0.5 | N | Y | Y | Y | Y | Y | Y | N | Y | Y | Y | Y |
| **OCF 48** | Hot Spring Lake | Intermediate | 7.41 | 1.31 | 0.7 | Y | Y | Y | Y | Y | Y | Y | N | Y | Y | Y | Y |
| **OCF 06** | Zelma Lake | Rain | 15.99 | 5.01 | 0.3 | Y | Y | Y | Y | Y | Y | Y | N | Y | Y | Y | Y |
| **OCF 29** | John Charlie Lake | Rain | 11.71 | 6.86 | 1.2 | Y | Y | Y | Y | Y | Y | Y | N | Y | Y | Y | Y |
| **OCF 37** | Tsii’vii Zhit | Rain | 8.84 | 5.14 | 1.2 | Y | Y | N | N | Y | Y | Y | N | Y | Y | N | Y |
| **OCF 38** | Husky Lake | Rain | 137.9 | 12.67 | 1.1 | N | Y | Y | N | Y | Y | Y | N | Y | N | Y | Y |
| **OCF 49** | Marten Lake | Rain | 10.75 | 1.15 | 1.2 | Y | Y | Y | Y | Y | Y | Y | N | Y | N | Y | Y |
| **Number of lakes with samplers retrieved per year** | | | | | | 7 | 13 | 14 | 12 | 14 | 14 | 14 | 0 | 14 | 11 | 13 | 14 |

Table S2: Diatom taxon names, authority names, and codes presented in the CCA ordination for the combined 2008-2009 data in Figure 2 and time-series graphs in Figure S1.

| Taxon Name | Authority | Code |
| --- | --- | --- |
| *Achnanthidium minutissimum* | (Kützing) Czarnecki 1994 | *AchMin* |
| *Amphipleura pellucida* | (Kützing) Kützing 1844 | *AmhPle* |
| *Asterionella formosa* | Hassall 1850 | *AstFor* |
| *Brachysira microcephala* | (Grunow) Compère 1986 | *BraMic* |
| *Cocconeis lineata* | Ehrenberg 1838 | *CocLin* |
| *Cymbella neocistula* | Krammer 2002 | *CymNeo* |
| *Diatoma tenuis* | C. Agardh 1812 | *DiaTen* |
| *Encyonema minutum* | (Hilse) D. G. Mann 1990 | *EncMin* |
| *Encyonema neogracile* | Krammer 1997 | *EncNeo* |
| *Encyonema obscurum* | (Krasske) D.G. Mann 1990 | *EncObs* |
| *Epithemia adnata* | (Kützing) Brébisson 1838 | *EpiAdn* |
| *Epithemia gibba* | (Ehrenberg) Kützing 1844 | *EpiGib* |
| *Eunotia bilunaris* | (Ehrenberg) Schaarschmidt 1881 | *EunBil* |
| *Eunotia species* | Species group | *EunSpp* |
| *Fragilaria tenera* | (W. Smith) Lange-Bertalot 1980 | *FraTen* |
| *Fragilariforma mesolepta* | (Rabenhorst) Kharitonov 2005 | *FrafMes* |
| *Frustulia amphipleuroides* | (Grunow) A. Cleve 1934 | *FruAmpl* |
| *Gomphonema acuminatum* | Ehrenberg 1832 | *GomAcu* |
| *Gomphonema angustum* | C. Agardh 1831 | *GomAng* |
| *Gomphonema capitatum* | Ehrenberg 1838 | *GomCap* |
| *Gomphonema gracile* | Ehrenberg 1838 | *GomGra* |
| *Gomphonema species 1* | Unknown species | *GomSp1* |
| *Gomphonema truncatum* | Ehrenberg 1832 | *GomTru* |
| *Navicula cryptocephala* | Kützing 1844 | *NavCrc* |
| *Navicula cryptotenella* | Lange-Bertalot 1985 | *NavCrn* |
| *Navicula radiosa* | Kützing 1844 | *NavRad* |
| *Nitzschia fonticola* | (Grunow) Grunow in Van Heurck 1881 | *NitFon* |
| *Nitzschia palea* | (Kützing) W. Smith 1856 | *NitPal* |
| *Nitzschia species* | Species group | *NitSpp* |
| *Nupela pennsylvanica* | (R. M. Patrick) Potapova 2011 | *NupPen* |
| *Pinnularia stomatophora* | (Grunow) Cleve 1895 | *PinStom* |
| *Rossithidium pusillum* | (Grunow) Round and Bukhtiyarova 1996 | *RosPus* |
| *Sellaphora pupula* | (Kützing) Mereschkovsky 1902 | *SelPup* |
| *Staurosirella pinnata* | (Ehrenberg) D. M. Williams & Round 1988 | *StalPin* |
| *Staurosira construens* | Ehrenberg 1843 | *StauCon* |
| *Tabellaria flocculosa* | (Roth) Kützing 1844 | *TabFlo* |
| *Tabularia fasciculata* | (C. Agardh) D. M. Williams & Round 1986 | *TabuFas* |
| *Ulnaria ulna* | (Nitzsch) Compère 2001 | *UlnUln* |

Table S3: Summary statistics and P-values and codes used for the water chemistry variables shown in the figures of results from the canonical correspondence analyses (CCA) of the combined 2008 and 2009 spatial data set of diatom percent abundances and environmental variables (Figures 2 and 3). The P-values were computed using 499 random Monte Carlo permutations. Also included are the codes for the physical and hydrological variables included as supplementary variables in the CCAs. The variable codes are used in the CCA ordination plots shown in Figures 2 and 3 of the manuscript.

| Variable name | Code | Variation Explained (%) | pseudo-F |  | P-value |
| --- | --- | --- | --- | --- | --- |
| **Water chemistry variables** |  |  |  |  |  |
| pH | pH | 11.0 | 9.8 |  | 0.002 |
| Alkalinity | Alk | 10.7 | 9.5 |  | 0.002 |
| Specific Conductivity | SpCond | 10.5 | 9.3 |  | 0.002 |
| [DIC] | DIC | 9.7 | 8.5 |  | 0.002 |
| [Mg] | Mg | 8.9 | 7.7 |  | 0.002 |
| [Ca] | Ca | 8.5 | 7.4 |  | 0.002 |
| [DOC] | DOC | 8.0 | 6.9 |  | 0.002 |
| [Na] | Na | 7.9 | 6.8 |  | 0.002 |
| [SO_4_] | SO4 | 4.7 | 3.9 |  | 0.002 |
| [TDP] | TDP | 4.4 | 3.6 |  | 0.002 |
| [SiO_2_] | SiO2 | 3.5 | 2.8 |  | 0.004 |
| [Cl] | Cl | 2.6 | 2.1 |  | 0.016 |
| [TP] | TP | 2.5 | 2.1 |  | 0.020 |
| [TN] | TN | 1.6 | 1.3 |  | 0.204 |
| [NH3] | NH3 | 1.5 | 1.2 |  | 0.236 |
| [Chlorophyll a] | Chl-a | 1.5 | 1.2 |  | 0.232 |
| [K] | K | 1.3 | 1.0 |  | 0.428 |
|  |  |  |  |  |  |
| **Physical and hydrological variables** | | |  |  |  |
| Evaporation:Inflow ratio | E/I | N/A | N/A |  | N/A |
| δ^18^O_I_ | δ18OI | N/A | N/A |  | N/A |
| Lake surface area | SA | N/A | N/A |  | N/A |


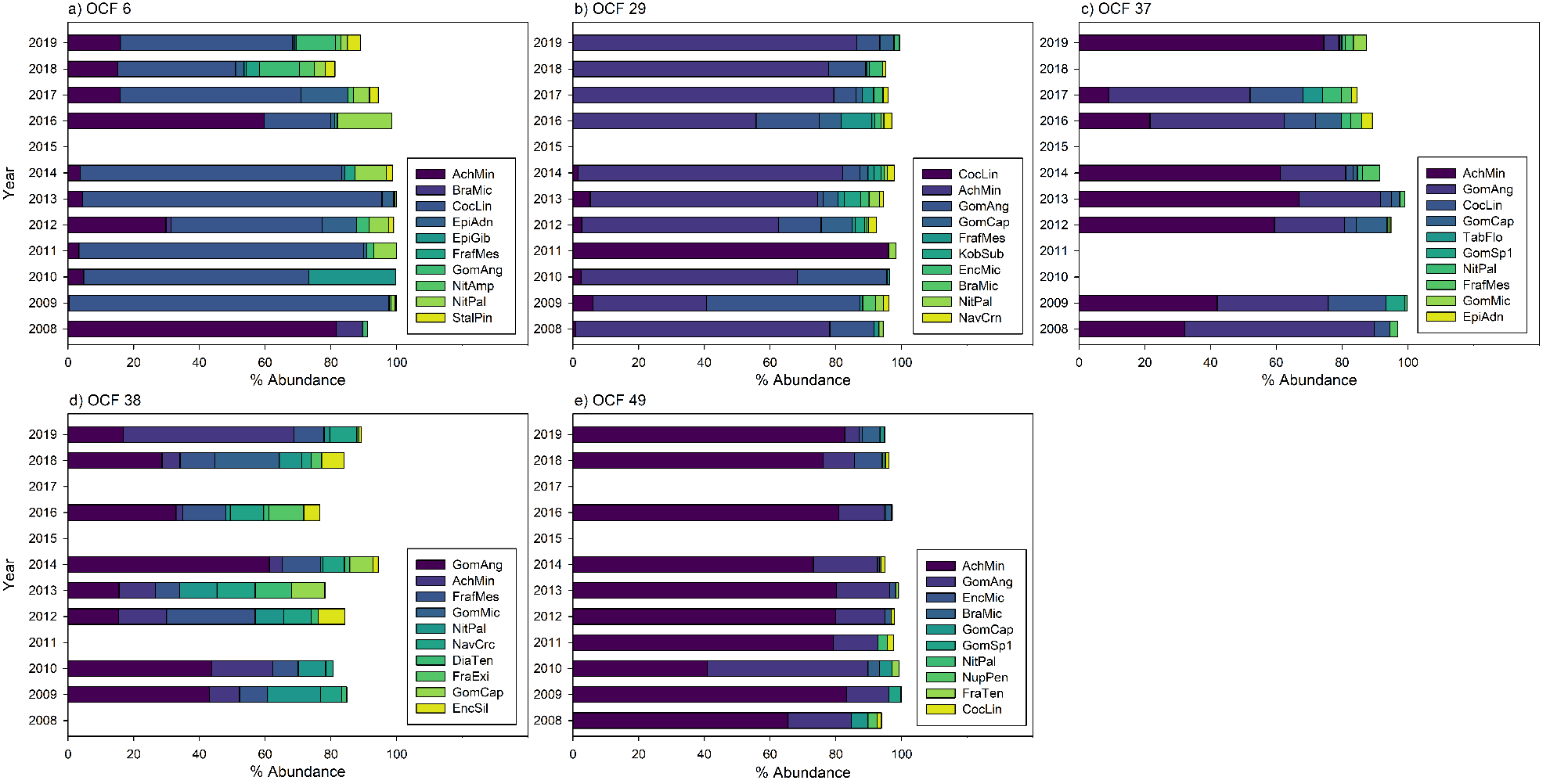


Supplemental Figure S1. Graphs showing temporal patterns of change in relative abundance of select diatom taxa accrued on artificial-substrate samplers during 2008-2019 in the long-term monitoring lakes that began the monitoring period in the rainfall-dominated category. Full names of the abbreviated diatom taxon codes are reported in Table S2.


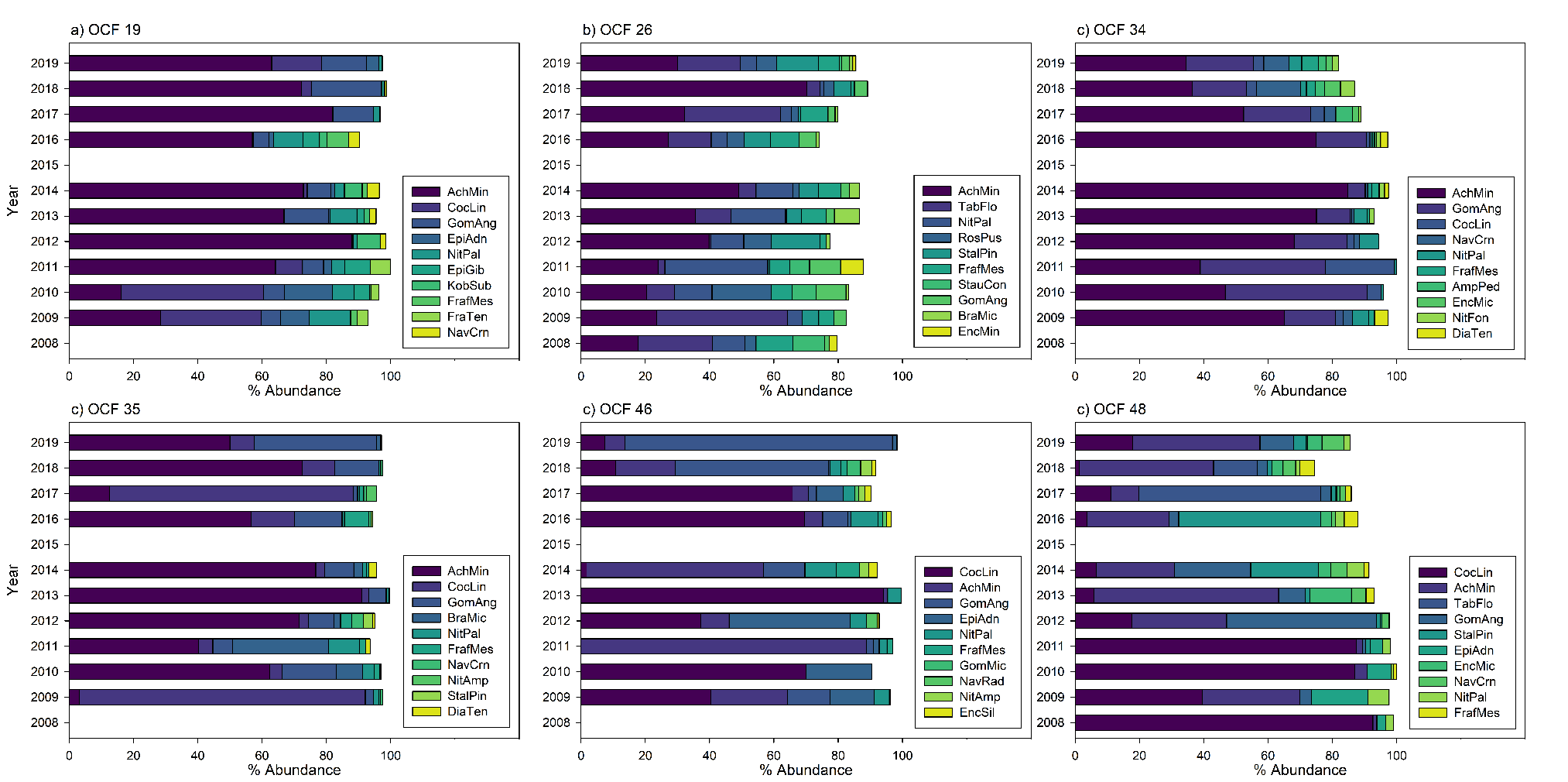


Supplemental Figure S2. Graphs showing temporal patterns of change in relative abundance of select diatom taxa accrued on artificial-substrate samplers during 2008-2019 in the long-term monitoring lakes that began the monitoring period in the intermediate category. Full names of the abbreviated diatom taxon codes are reported in Table S2.


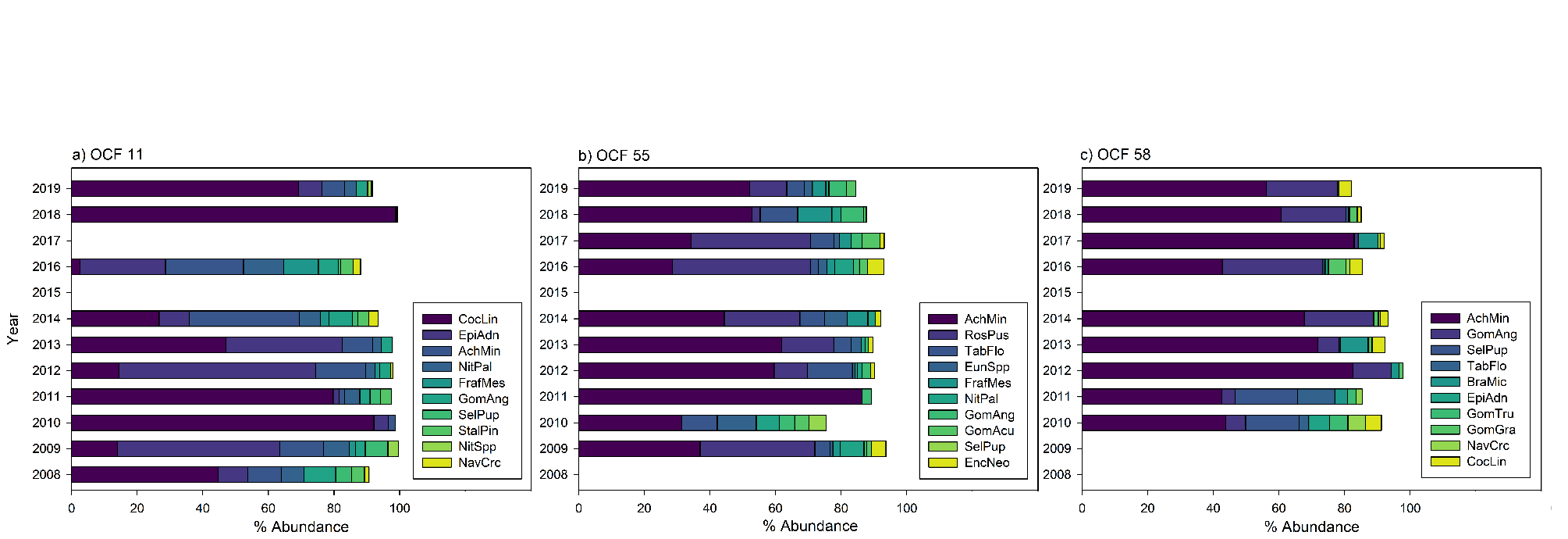


Supplemental Figure S3. Graphs showing temporal patterns of change in relative abundance of select diatom taxa accrued on artificial-substrate samplers during 2008-2019 in the long-term monitoring lakes that began the monitoring period in the snowmelt-dominated category. Full names of the abbreviated diatom taxon codes are reported in Table S2.
